# Radiobiological and dosimetric comparison of 60Co versus 192Ir high-dose-rate intracavitary-interstitial brachytherapy for cervical cancer

**DOI:** 10.1186/s13014-022-02170-8

**Published:** 2022-12-13

**Authors:** Aiping Wen, Xianliang Wang, Bingjie Wang, Chuanjun Yan, Jingyue Luo, Pei Wang, Jie Li

**Affiliations:** 1 School of Medicine, University of Electronic Science and Technology of China, Chengdu, 610054 China; 2 Department of Radiation Oncology, Sichuan Cancer Hospital and Institute, Sichuan Cancer Center, Radiation Oncology Key Laboratory of Sichuan Province, Chengdu, 610041 China; 3 Department of Health Technology and Informatics, The Hong Kong Polytechnic University, Hongkong, 999077 China; 4 Department of Oncology, The Affiliated Hospital of Southwest Medical University, Luzhou, 646000 Sichuan China

**Keywords:** 60Co, 192Ir, HDR brachytherapy, IC-ISBT, Cervical cancer

## Abstract

**Background:**

High-dose-rate (HDR) intracavitary-interstitial brachytherapy (IC-ISBT) is an effective treatment for bulky, middle, and advanced cervical cancer. In this study, we compared the differences between 60Co and 192Ir HDR IC-ISBT plans in terms of radiobiological and dosimetric parameters, providing a reference for clinical workers in brachytherapy.

**Methods:**

A total of 30 patients with cervical cancer receiving HDR IC-ISBT were included in this study, and IC-ISBT plans for each individual were designed with both 60Co and 192Ir at a prescribed dose of CTV D90 = 6 Gy while keeping the dose to OARs as low as possible. Physical dose and dose–volume parameters of CTV and OARs were extracted from TPS. The EQD2, EUBED, EUD, TCP, and NTCP were calculated using corresponding formulas. The differences between the 60Co and 192Ir IC-ISBT plans were compared using the paired t-test.

**Results:**

In each patient's 60Co and 192Ir IC-ISBT plan, the average physical dose and EQD2 of 60Co were lower than those of 192Ir, and there were statistically significant differences in D2cc and D1cc for the OARs (*p* < 0.05); there were statistically significant differences in D0.1 cc for the bladder (*p* < 0.05) and no significant differences in D0.1 cc for the rectum or intestines (*p* > 0.05). The EUBED ratio (60Co/192Ir) at the CTV was mostly close to 1 when neither 60Co or 192Ir passed their half-lives or when both passed two half-lives, and the difference between them was not significant; at the OARs, the mean value of 60Co was lower than that of 192Ir. There was no statistical difference between 60Co and 192Ir in the EUD (93.93 versus 93.92 Gy, *p* > 0.05) and TCP (97.07% versus 97.08%, *p* > 0.05) of the tumors. The mean NTCP value of 60Co was lower than that of 192Ir.

**Conclusions:**

Considering the CTV and OARs, the dosimetric parameters of 60Co and 192Ir are comparable. Compared with 192Ir, the use of 60Co for HDR IC-ISBT can ensure a similar tumor control probability while providing better protection to the OARs. In addition, 60Co has obvious economic advantages and can be promoted as a good alternative to 192Ir.

## Introduction

Cervical cancer is the fourth leading cause of cancer death among women worldwide, and morbidity and mortality continue to increase each year in regions with poor access to prevention and screening measures [1, 2]. Cervical cancer has high radiosensitivity and is often treated with external beam radiotherapy (EBRT) and intracavitary/interstitial brachytherapy (IC/ISBT). For bulky, middle, and advanced cervical cancer, the treatment coverage of EBRT combined with ICBT has certain limitations [3–5], while in ISBT, an insertion needle can be placed according to the tumor shape and then used in combination with an intracavitary applicator to ensure that the central part of the cervix receives a high dose, covering the target area accurately and conformally so as to achieve more effective treatment. At present, cervical cancer brachytherapy is mainly based on a high dose rate (HDR). The two radioactive sources recommended in ICRU report 89 are 192Ir and 60Co. Previously, due to the large geometric size of 60Co and its limited clinical use, 192Ir occupied most of the market share. After continuous technical improvements, the geometry of miniaturized 60Co sources has become comparable to that of 192Ir sources. The 60Co (1.25 MeV) radiation source has higher gamma energy than 192 Ir (0.38 MeV), which indicates that a higher standard of medical protection is required for the use of 60Co. There are concerns that 60Co may have some damaging consequences distinct from those of 192Ir, such as excessive doses to normal organs and tissues and potentially increased toxicity. However, because 60Co has a long half-life (about 5 years) compared with 192Ir (74 days), it has obvious advantages in terms of human resources, logistics, and the economy. For developing countries, it is undoubtedly more cost-effective to choose a 60Co source [6, 7]. There are few reports on the differences between using 60Co and 192Ir for intracavitary-interstitial brachytherapy (IC-ISBT). Therefore, this study compares the differences in the physical dose and radiobiological effects on tumors and organs at risk (OARs) using these two radiation sources in IC-ISBT, conducting an analysis of the findings to provide a reference for clinical workers in brachytherapy.

## Methods and materials

### Patient selection and applicator insertion

Thirty patients diagnosed with cervical cancer (stage IB2 to IVA according to FIG.O 2018, squamous cell carcinoma) in Sichuan Cancer Hospital from January 1, 2021, to October 31, 2021, were selected for retrospective analysis. All patients underwent pelvic EBRT (45 Gy) and IC/IC-ISBT (6 Gy × 5 fractions). Before ISBT, the first CT scan was performed in a molded fixed position, ranging from the plane of the iliac spine to 2 cm below the vaginal opening, and the slice thickness was 3 mm. The insertion was immediately performed as a routine operation by three experienced clinicians, using a ProGuide sharp needle (Part# 189.601) and a Henschke titanium applicator (Part#110.437), and comprehensive evaluation of the patient's tumor location and size was performed to determine the required number of needles and the depth of insertion into the tumor tissue. A second CT scan was performed immediately after insertion, and the image data were transmitted to the Oncentra brachytherapy planning system. Before each CT scan, the catheter should be opened to empty the urine, and then 100 mL normal saline should be injected into the bladder to ensure that the filling state of the bladder remains unchanged. All patients were transported using a special transfer bed to prevent both position changes and movement of the implantation needle and metal applicator.

### Contouring and treatment planning

We used the Oncentra treatment planning system (Elekta AB, Stockholm, Sweden, version 4.3) to design two brachytherapy plans on the CT for each patient, one using 60Co and one using 192Ir. The tumor and the OARs (including the bladder, rectum, sigmoid, and intestines) were outlined by oncologists according to the recommendations of the GEC-ESTRO working group. Then, a physicist performed applicator reconstruction, referring to the ICRU recommendations. The step length of the radiation source was 5 mm, the length of the metal applicator was 150 cm with an OFFSET value (the distance from the top of the applicator to the first dwelling position) of -0.6 cm, and the needle length was 124 cm with an OFFSET value of − 0.4 cm. All plans were optimized using the hybrid inverse planning optimization (HIPO) algorithm. When more needles are used, the HIPO algorithm can provide a better dose distribution in the high-dose region [8]. The dwell time gradient ratio (DTGR) was 0.6. The target parameters were continuously optimized so that the CTV D90 in the 60Co and 192Ir treatment plans of all patients reached 600 cGy (whereby the difference did not exceed 1 cGy), and the OAR D2cc value was as low as possible. The total dose of EBRT and HDR BT should meet the requirements of HR-CTV ≥ 85–90 Gy EQD2. For patients with severe disease, these requirements are HR-CTV D90 ≥ 87 Gy EQD2, rectum D2cc ≤ 65–75 Gy EQD2, sigmoid D2cc ≤ 70–75 Gy EQD2, and bladder D2cc ≤ 80–90 Gy EQD2 [9].

### Parameters calculation

The use of the linear quadratic (LQ) model has been validated, and it has become the dominant model for radiobiology. The model proposes the use of α/β values in order to evaluate the radiotherapy sensitivity of tumors and normal tissues to fractionated irradiation doses and dose rate changes; increasing the dose of a single fraction of irradiation and reducing the number of fractions will significantly increase the radiation damage to late-reacting tissues [10]. The design of HDR BT plans based on the LQ model helps clinicians to conduct comprehensive evaluation of applying a single irradiation dose and determining the required number of fractions for patients. We extracted dose–volume data (D0.1 cc, D1cc, and D2cc) of HR-CTV and the OARs (the bladder, rectum, and intestines) from each patient's 60Co and 192Ir ISBT plans and strictly normalized the CTV D90 of all patients' ISBT plans to 600 cGy. The physical dose and equivalent dose in 2 Gy/f (EQD2) of the OARs were calculated for D0.1 cc, D1cc, and D2cc using Eq. (1).

1 $$EQD_{2} = D\frac{d + \alpha /\beta }{{2 + \alpha /\beta }}$$

where D is the total irradiation dose (fraction *n* × single irradiation dose d). The Groupe Europeen de Curietherapie and the European Society for Therapeutic Radiology and Oncology (GEC-ESTRO) working group recommends an α⁄β of 10 for cervical cancer (CTV) and 3 for normal tissues (OARs).

The biologically effective dose (BED) can be used for comparisons between different fraction schemes. In addition, in the LQ model, the G factor takes into account the time and dose changes, which can better describe the dose rate and cell sublethal damage repair ability through the effect on the *β* component [11]. The expressions of BED and the G factor are as follows:

2 $$BED = D\left( {1 + \frac{D}{\alpha /G\beta }} \right)$$

3 $$G = \left( {\frac{2}{{d^{2} }}} \right)\mathop \smallint \limits_{0}^{T} {\text{du}} I\left( u \right) \mathop \smallint \limits_{0}^{T} {\text{dw}} I\left( w \right) H\left( {u - w} \right) \times {\text{exp}}\left( { - \left( {u - w} \right)/T_{1/2} } \right)$$

where *d* is the dose of a single fraction; I(t) is the dose rate at time t; T_1/2_ is the repair half-time; and H(u-w) is the Heaviside function, defined as unity for u ≥ w and zero otherwise.

Both BED and EQD2 calculations assume a homogeneous dose distribution, but within the range of HDR BT treatment, there is anatomical heterogeneity in the irradiated tissue. Because each reference point is different from the radiation source, the dose rate and dose deposition differ, the dose distribution has obvious inhomogeneity, and the biological effects on the different parts also vary. Therefore, BED and EQD2 do not describe the radiobiological effects of HDR BT with sufficient precision. The concept of equivalent uniform biologically effective dose (EUBED) was thus introduced for improved description of the role of radiobiological effects arising from dose inhomogeneity in clinical outcomes [12–15].

4 $${\text{EUBED}} = - \frac{1}{{\alpha_{j} }}\ln \left( {\mathop \sum \limits_{i = 1}^{N} v_{i} e^{{ - \alpha \cdot {\text{BED}}}} } \right)$$

The parameter range of ${\alpha }_{j}$ is 0.05–0.5 Gy^−1^.

Mathematical models of normal tissue complication probability (NTCP) and tumor control probability (TCP) have been proposed for better optimization and evaluation of HDR BT plans. To address inhomogeneous dose distribution, Niemierko proposed the concept of an equivalent uniform dose (EUD) [16, 17], which refers to a uniform dose with the same probability of radiobiological damage as that produced by an inhomogeneous dose distribution. These can be calculated from the dose–volume data of the tumors and OARs, which are extracted from the TPS using the following expressions:

5 $$EUD = \left( {\mathop \sum \limits_{i = 1} \left( {V_{i} D_{i}^{a} } \right)} \right)^{\frac{1}{a}}$$

where a is a specific unitless model parameter, *V*_*i*_ is unitless, *D*_*i*_ is the dose received by the voxel volume at *i*, and the sum of all partial volumes *V*_*i*_ is equal to 1. The local control rate of the tumor is likely to depend on the volume receiving the smallest dose, which represents the place with the highest clonal survival of tumor cells. Therefore, the EUD of the tumor will be close to the minimum dose, and parameter a should be a large negative number. In normal tissues with tandem structures, the EUD will be close to the maximum dose, and parameter a is usually a large positive number [18–20]. a_tumor_ = − 10,a_bladder_ = 2,a_rectum_ = 8.33,a_intestines_ = 6。Based on the concept of EUD, Niemierko proposed the TCP/NTCP model:

6 $${\text{TCP}} = \frac{1}{{1 + \left( {\frac{{{\text{TCD}}_{50} }}{{{\text{EUD}}}}} \right)^{{4\gamma_{50} }} }}$$

7 $${\text{NTCP}} = \frac{1}{{1 + \left( {\frac{{{\text{TD}}_{50} }}{{{\text{EUD}}}}} \right)^{{4\gamma_{50} }} }}$$

We used the published code of Gay, H. A. et al. for calculating TCP/NTCP[20], which takes into account the total dose of radiation therapy (EBRT 45 Gy + IC-ISBT 6 Gy × 5 fractions). TCD_50_ is the irradiation dose received at a tumor control rate of 50%, ${\gamma }_{50}$=3; TD_50_ is the dose tolerated by normal tissue at a complication rate of 50% for OARs (the bladder, rectum, and intestines), ${\gamma }_{50}$=4[21, 22].

### Statistics

We extracted the dose–volume data from the TPS of the CTV and OARs (the bladder, rectum, and small bowel) at D2cc, D1cc, and D0.1 cc for all patients with 60Co versus 192Ir BT plans. The differences in brachytherapy between the two sources, 60Co and 192Ir, were evaluated by comparing their dosimetric and radiobiological parameters. We used the paired-sample t-test to evaluate these differences on the premise that the samples met an approximately normal distribution, and the difference was statistically significant at *p* < 0.05. All statistical analyses were performed using IBM SPSS Statistics, version 22. (Table 1).

**Table 1:** Constraints for the HIPO algorithm

Name	Min weight	Min value(cGy)	Max value(cGy)	Max weight
HRCTV	100	600	1200	1
Bladder			400	100
Intestines			400	100
Rectum			400	100
Sigmoid			400	100
Normal tissue			1200	1

## Results

Table 2 shows the mean ± SD of the dose values of D2cc, D1cc, and D0.1 cc in the OARs (the bladder, rectum, and intestines) for 60Co and 192Ir for a single ISBT irradiation dose in 30 patients. As can be seen in Table 2, as the cumulative dose values of each OAR gradually increased from D2cc to D0.1 cc, the difference between the mean values of the 60Co and 192Ir plans gradually decreased, and the dose values of the 60Co plans were all lower than those of the 192Ir plans. At D2cc and D1cc, the differences between the physical doses of 60Co versus 192Ir were statistically different in the bladder, rectum, and intestines (p < 0.05); at D0.1 cc, the differences when comparing 60Co and 192Ir in the bladder remained statistically significant (*p* < 0.05), whereas there was no statistically significant difference (*p* > 0.05) in the rectum (*p* = 0.240) or intestines (*p* = 0.298). In the 60Co and 192Ir plans, the difference in the mean physical dose to the bladder was the largest among the OARs: at D2cc, the difference was 0.11 Gy, and 60Co was 2.29% lower than 192Ir; at D1cc, the difference was 0.11 Gy, and 60Co was 2.16% lower than 192Ir; and at D0.1 cc, the difference was 0.1 Gy, and 60Co was 1.73% lower than 192Ir.

**Table 2:** Differences between 60Co and 192Ir according to physical dose to OARs (the bladder, rectum, and intestines)

OARs	Mean ± SD (Gy)		Mean difference	Percentage mean difference	*p*
	D2cc				
	60Co	192Ir			
Bladder	4.70 ± 0.29	4.81 ± 0.28	− 0.11	− 0.0229	0.000
Rectum	4.41 ± 0.68	4.49 ± 0.68	− 0.08	− 0.0178	0.000
Intestines	3.57 ± 1.12	3.66 ± 1.13	− 0.09	− 0.0246	0.000

OARs	Mean ± SD (Gy)		Mean difference	Percentage mean difference	*p*
	D1cc				
	60Co	192Ir			
Bladder	4.99 ± 0.30	5.10 ± 0.29	− 0.11	− 0.0216	0.000
Rectum	4.82 ± 0.66	4.90 ± 0.65	− 0.08	− 0.0163	0.018
Intestines	3.96 ± 1.17	4.04 ± 1.18	− 0.08	− 0.0198	0.000

OARs	Mean ± SD (Gy)		Mean difference	Percentage mean difference	*p*
	D0.1 cc				
	60Co	192Ir			
Bladder	5.67 ± 0.39	5.77 ± 0.38	− 0.10	− 0.0173	0.003
Rectum	5.71 ± 0.67	5.77 ± 0.66	− 0.06	− 0.0104	0.240
Intestines	5.01 ± 1.43	5.05 ± 1.43	− 0.04	− 0.0079	0.298

Table 3 presents the mean ± SD of the EQD2 of the 30 patients, and they were calculated using formula (1). The calculation results are based on the physical dose, so the data distribution trend in Table 3 is the same as that in Table 2. At D2cc, D1cc, and D0.1 cc of each OAR, EQD2 was lower in the 60Co than in the 192Ir plans. At D2cc and D1cc, for 60Co versus 192Ir, there was a statistically significant difference in the physical dose for each OAR (*p* < 0.05); at D0.1 cc, there was a statistically significant difference in the bladder (*p* < 0.05), while there was no statistically significant difference in the rectum (*p* = 0.265) or the intestines (*p* = 0.354) (*p* > 0.05). In the plans of the two radioactive sources, the difference in the mean EQD2 of the bladder was the largest among the OARs.

**Table 3:** Differences between 60Co and 192Ir according to EQD2 of OARs (the bladder, rectum, and intestines)

OARs	Mean ± SD (Gy)		Mean difference	Percentage mean difference	*p*
	D2cc				
	60Co	192Ir			
Bladder	7.24 ± 0.72	7.53 ± 0.70	− 0.29	− 0.0385	0.000
Rectum	6.62 ± 1.60	6.82 ± 1.62	− 0.20	− 0.0293	0.000
Intestines	4.94 ± 2.08	5.12 ± 2.13	− 0.18	− 0.0352	0.000
	D1cc				
	60Co	192Ir			
Bladder	7.99 ± 0.77	8.29 ± 0.75	− 0.30	− 0.0362	0.000
Rectum	7.62 ± 1.63	7.82 ± 1.65	− 0.20	− 0.0256	0.023
Intestines	5.78 ± 2.30	5.96 ± 2.36	− 0.18	− 0.0302	0.000
	D0.1 cc				
	60Co	192Ir			
Bladder	9.85 ± 1.12	10.15 ± 1.09	− 0.30	− 0.0296	0.004
Rectum	10.04 ± 1.93	10.21 ± 1.93	− 0.17	− 0.0167	0.265
Intestines	8.42 ± 3.36	8.52 ± 3.40	− 0.10	− 0.0117	0.354

**Fig. 1:**
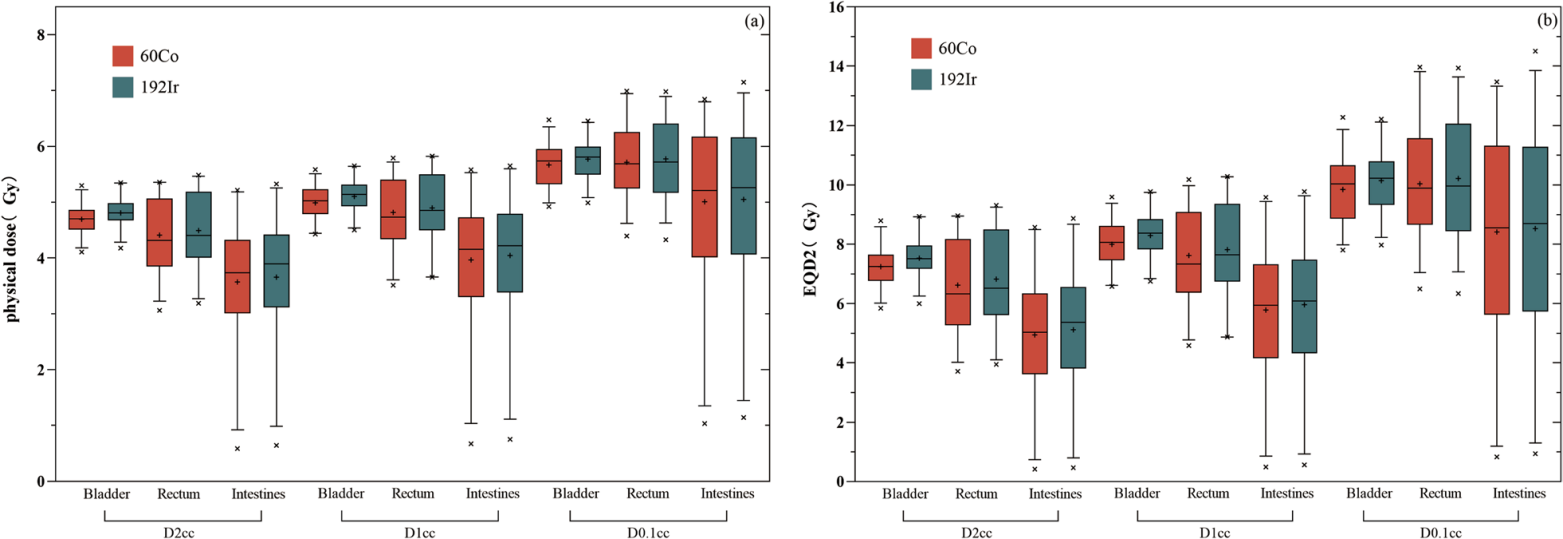
A box plot of physical dose **a** and EQD2 **b** received by the bladder, rectum, and intestines for 30 patients. Boxes show median value and 5–95% range, cross symbols indicate maximum and minimum values, and the plus sign indicates the mean value

Figure 1 displays a box plot of the physical dose and EQD2 received by the bladder, rectum, and intestines of the 30 patients. The boxes show the median value and 5%-95% range, the cross symbols indicate the maximum and minimum values, and the plus sign indicates the mean value. Figure 1 shows that, in general, the intestines receive a lower dose than the bladder and rectum, and there are large individual differences among the different patients. The average value of 60Co is lower than that of 192Ir, and the difference between them is small.

**Fig. 2:**
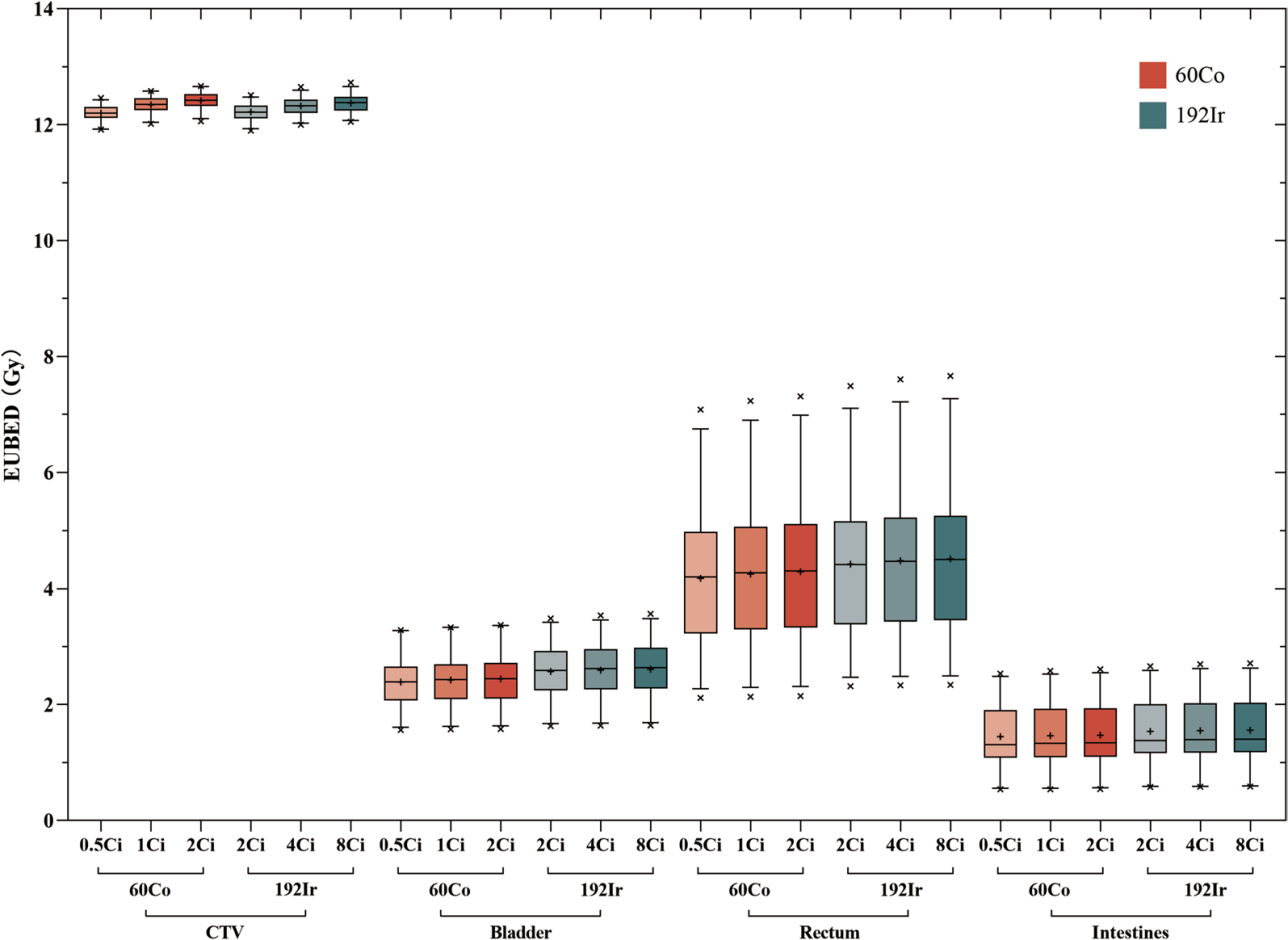
A box plot of EUBED values for the CTV, bladder, rectum, and intestines at different activity levels for 60Co and 192Ir. Boxes show median value and 5–95% range, cross symbols indicate maximum and minimum values, and the plus sign indicates the mean value

**Fig. 3:**
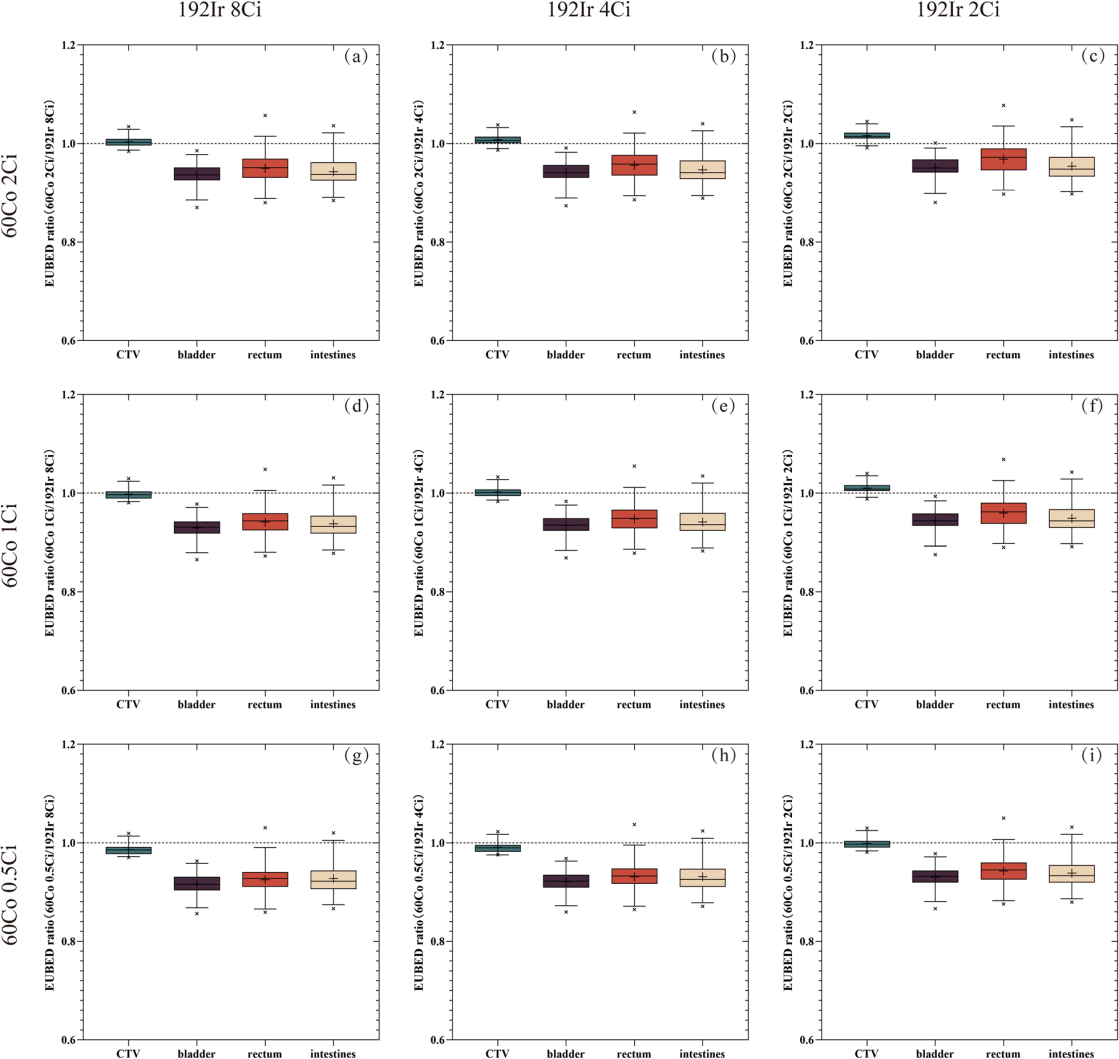
The EUBED ratio produced by 60Co and 192Ir at different activity levels

The half-lives of 60Co and 192Ir are quite different. Considering the decay of the radioactive source, we calculated the EUBED values generated by the IC-ISBT plans of the 30 patients at different activity levels. Figure 2 shows the results of the EUBED calculation for all patients, and Table 4 shows the mean ± SD EUBED values for the CTVs and OARs. As can be seen in the above graphs, the bioeffective doses considering the CTV and OARs gradually decrease with the decay of the radioactive sources. In addition, we calculated the EUBED ratios (60Co/192Ir) at different activity levels (Fig. 3). Regarding CTV, when neither 60Co or 192Ir pass their half-life (as in Fig. 3. (a), (b), (d), (e)), or when both pass two half-lives (as in Fig. 3. (h)), their EUBED ratios are mostly close to 1, and the difference between them is not obvious; when 60Co is 1 or 2 Ci and 192Ir is 2 Ci, as in Fig. 3. (c) and (f), their EUBED ratios are mostly greater than 1, and 60Co produces a higher EUBED; when 192Ir is 8 or 4 Ci and 60Co is 0.5 Ci, as in Fig. 3. (g) and (h), their EUBED ratios are mostly less than 1, and 192Ir produces a higher EUBED.

**Table 4:** EUBED for the CTV, bladder, rectum, and intestines at different activity levels for 60Co and 192Ir

		CTV/Gy	Bladder/Gy	Rectum/Gy	Intestines/Gy
*60Co*	2 Ci	12.42 ± 0.15	2.44 ± 0.47	4.29 ± 1.30	1.47 ± 0.56
	1 Ci	12.34 ± 0.14	2.42 ± 0.47	4.25 ± 1.28	1.46 ± 0.55
	0.5 Ci	12.20 ± 0.14	2.39 ± 0.45	4.18 ± 1.25	1.45 ± 0.54
*192Ir*	8 Ci	12.37 ± 0.16	2.61 ± 0.50	4.51 ± 1.34	1.56 ± 0.57
	4 Ci	12.32 ± 0.16	2.60 ± 0.50	4.48 ± 1.33	1.55 ± 0.57
	2 Ci	12.22 ± 0.15	2.57 ± 0.49	4.42 ± 1.30	1.54 ± 0.56

Table 5 shows the mean EUD for the CTV and OARs for the 30 patients. The statistical results are based on the total dose of EBRT (45 Gy) and IC-ISBT (6 Gy × 5 fractions). As can be seen in the Table 5, there was no statistically significant difference in EUD between the 60Co and 192Ir plans in tumors (*p* > 0.05), while there was a statistically significant difference in the OARs (*p* < 0.05), with 60Co being 1.26% lower than 192Ir in the bladder, 1.31% lower in the rectum, and 0.82% lower in the intestines.

**Table 5:** EUD values of 60Co and 192Ir for the CTV, bladder, rectum, and intestines

	Mean ± SD (Gy)		Mean difference	Percentage mean difference	*p*
	60Co	192Ir			
Tumor	93.93 ± 1.52	93.92 ± 1.60	0.01	0.0001	0.974
Bladder	54.76 ± 1.89	55.46 ± 1.97	− 0.70	− 0.0126	0.000
Rectum	64.01 ± 4.21	64.86 ± 4.17	− 0.85	− 0.0131	0.000
Intestines	51.87 ± 2.63	52.30 ± 2.73	− 0.43	− 0.0082	0.000

Table 6 shows the mean TCP and NTCP values in the 60Co and 192Ir plans for the 30 patients. The tumor control probability values of 60Co and 192Ir both reached 97% and were not statistically significantly different (*p* > 0.05). The NTCP of the bladder (0.27 for 60Co and 0.33 for 192Ir) was the lowest of all the OARs; the use of 60Co was 18.18% lower than that of 192Ir, and there was a statistical difference between the two sources (*p* < 0.05). The NTCP values of 60Co and 192Ir were significantly different in the rectum and intestines (*p* < 0.05), where 60Co was 16.87% and 8.23% lower than 192Ir, respectively.

**Table 6:** Mean tumor control probability and normal tissue complication probability in 60Co and 192Ir plans for 30 patients

		Mean ± SD (%)		Mean difference	Percentage mean difference	*p*
		60Co	192Ir			
TCP	Tumor	97.09 ± 0.58	97.08 ± 0.97	0.01	0.0001	0.861
NTCP	Bladder	0.27 ± 0.15	0.33 ± 0.18	− 0.06	− 0.1818	0.000
	Rectum	4.14 ± 4.17	4.98 ± 5.3	− 0.84	− 0.1687	0.030
	Intestines	29.88 ± 15.78	32.56 ± 16.68	− 2.68	− 0.0823	0.000

## Discussion

HDR BT is currently an important component of cervical cancer treatment. For bulky, middle, and advanced cervical cancer, IC-ISBT can better conform to the tumor area and has excellent therapeutic effects [23]. The dose distribution of brachytherapy is characterized by a high dose close to the center of the radiation source, rapid drop in the dose to the surrounding area, and uneven distribution of the actual brachytherapy dose due to heterogeneity of the human anatomical structure. The ICRU recommends the use of rectal and bladder reference points for the dose assessment of normal organ tissues in HDR BT; however, with the continuous development of 3D BT technology, studies have confirmed that a DVH dose assessment based on CTV and OARs often differs from those based on ICRU reference points. DVH-based dose assessment results are more conformal and reliable, while TCP and NTCP calculations can be performed based on the results, which is conducive to individually designed radiotherapy plans and improved tumor control rates [24–26].

Radiation sources 60Co and 192Ir have similar dose distribution trends. Many previous studies have demonstrated that the dose parameters of the HR-CTV, bladder, and rectum in brachytherapy plans of 60Co are comparable to those of 192Ir. Although the dose line distribution in the cephalocaudal direction is more prominent for 60Co than for 192Ir, they essentially become the same after optimization of the radiotherapy plan by a physicist, with the difference reduced to the extent that it is no longer clinically significant [27–30]. In this study, we selected 30 patients and designed two radiotherapy plans of 60Co and 192Ir for each individual patient, and all plans were given the prescribed dose of CTV D90 6 Gy. The data extracted from TPS were processed using calculations, and the physical dose values of CTV D90 and EQD2 were equal for both the 60Co and 192Ir plans. Table 2 shows that there is a statistically significant difference between the two sources in the OAR D2cc and D1cc doses, with 60Co producing a lower mean physical dose than 192Ir. This may be due to the steeper dose gradient of 60Co. At distances less than 1 cm from the source, the 60Co source produced higher dose rates and, therefore, higher cumulative physical dose values, while at distances greater than 1 cm from the source, the 192Ir source produced higher dose rates [31]. Moreover, the bladder and rectum had higher doses than the intestines due to their location being closer to the source of radiation. It has previously been demonstrated that 60Co produces a larger hot spot than 192Ir in the CTV of tumors and that the dose rate falls more rapidly as the distance from the source increases [32–34]. Compared to the published results, the internal, external, and obturator lymph nodes etc., (locations far away from the center of the source) receive a lower dose when using 60Co for ICBT [35]. These OARs are located relatively far away from the source of radiation and, therefore, receive less exposure. The results for D2cc, D1cc, and D0.1 cc not only represent dose–volume data in treatment plans but also allow for an overall dose assessment and complication prediction for OARs [36–38]. Based on the analysis of the experimental data, we speculate that the use of 60Co in IC-ISBT will provide better protection to OARs than 192Ir.

Cervical cancer has a strong self-healing ability. Although a large number of cells may proliferate in the interval between two treatments, hypoxic cells may also increase, resulting in increased radioresistance. However, because the dose distribution of interstitial implants may be more adequate, the dose of brachytherapy is high enough to overcome the increased radiation resistance caused by hypoxia, thereby maintaining a good tumor control rate [39, 40]. High dose rates of brachytherapy above 12 Gy/h are very beneficial for tumor tissue regression. It can be seen that, in order to achieve better therapeutic effects, HDR BT has certain requirements in terms of the activity of the radioactive source. It can be seen in Fig. 3 that the dose rates of the two radioactive sources, whether 60Co or 192Ir, show the same trend, and a decrease in activity leads to a decrease in EUBED. In the case of higher activity of the radioactive source, there was no significant difference in the EUBED values produced by the two sources at the CTV. In the OARs, the EUBED of 60Co was significantly lower than that of 192Ir, which may be due to the fact that the OARs received a lower radiation dose and were farther away from the radioactive source in response to 60Co, thus resulting in a lower dose rate. The half-life of 60Co is about 5.27 years, and the half-life of 192Ir is about 74 days. In order to ensure the therapeutic effect, 192Ir should be replaced about every six months, and 60Co thus has obvious advantages from the perspective of economic efficiency.

When designing a radiotherapy plan, in addition to optimization based on the distribution of physical doses, another important evaluation method is based on TCP and NTCP. In the treatment of cervical cancer, TCP is positively correlated with irradiation dose. Increasing the dose rate and shortening the total treatment time can improve TCP, but at the same time, the risks of normal tissue damage and late complications increase with dose [41]. In this study, we chose the model proposed by Niemierko, which is calculated based on EUD (Eq. 5). The calculation for EQD2 in the previous paper was based on D90 and did not take into account the non-uniform dose distribution in brachytherapy, so it is possible that the calculated value was lower than the actual value. The EUD weighting factor was introduced in Eq. 5, which can better describe the tumor area with a non-uniform dose distribution and has a better fitting ability. In Table 5, the EUD values in the tumors for 60Co and 192Ir are not statistically different, indicating that the choice of radiation source has no significant effect on the equivalent uniform dose distribution at the CTV; for the OARs, the use of 60Co compared with 192Ir produces lower mean EUD values. However, since the actual physical dose distribution of the OARs is more heterogeneous than the tumor area in HDR BT, routine EUD calculation and evaluation of the OARs are not recommended, and D2cc, D1cc, or D0.1 cc is recommended instead [36]. Table 2 shows that for TCP, the use of 60Co does not cause results that are significantly different from those of 192Ir; in the OARs with statistical differences, the mean NTCP is slightly lower for 60Co than for 192Ir, especially regarding the NTCP of the bladder, which is reduced by 18.18%. Intestinal cells have a higher ionizing radiation sensitivity and a higher probability of complications (enterocolitis, intestinal fistula, etc.) after radiotherapy, and the predicted outcomes of 60Co and 192Ir have the same trend of distribution, so there is no disadvantage of 60Co compared to 192Ir in terms of disease control. In published clinical trials, there are results showing that patients with cervical cancer treated with 60Co HDR ISBT have similar survival and toxicity outcomes to those treated with 192Ir [42–44]. However, there is still a lack of large sample sizes and randomized clinical trials using 60Co and 192Ir for IC-ISBT in patients with cervical cancer to establish definitive conclusions. Therefore, further research is needed to investigate the differences in clinical outcomes, such as the local control rate and survival time, between the two radioactive sources.

In this study, data from 30 patients treated with a single ISBT irradiation dose were selected to investigate the radiobiological differences between 60Co and 192Ir, but in the actual clinical treatment process, the biological characteristics of tumors and normal tissues are not only affected by a single irradiation. The following five major biological factors underlie the rationale for fractionated radiation therapy: radiosensitivity, repair, regeneration, redistribution, and reoxygenation. After each radiotherapy, the tumor tissue regression and patient's physical tolerance will influence the next IC-ISBT. An increase in tumor hypoxic cells after treatment also increases radioresistance to the next treatment. Residual cells after irradiation may “repopulate”, and if a sufficiently large number of tumor cells repopulate in the interval between fractions, the treatment may fail. The repair of sublethal damage to normal tissues may not be completed in time, so the control of the tumor cannot be separated from the time of the entire radiotherapy [45]. In the study conducted by Dayyani, M. et al., considering the influence of RBE correction and a patient's full treatment regimen, the BED produced by 60Co was lower, so it was recommended that the prescribed dose be increased by 4% when using a 60Co source; the EQD2 of OARs after dose escalation was close to or even lower than that of 192Ir [33]. However, our study was mainly conducted to compare the physical dose and radiobiological differences in a single IC-ISBT irradiation dose with regard to the choice of radioactive source, so the other treatments, including the EBRT that was performed and the remaining four ISBTs, were treated uniformly and were simplified. The statistical results can provide a reference for brachytherapy workers. In order to obtain higher level clinical evidence, this comparative trial needs to be further extended to address the aforementioned issues.

## Conclusion

By comparing the results of 192Ir and 60Co IC-ISBT plans, it can be concluded that, after constant optimization and given the same constraints, there is no significant difference in the radiobiological effect parameters and TCP at the CTV between the two radioactive sources. For the OARs, the average physical dose and EQD2 of 60Co in D2cc were lower than those of 192Ir, the average NTCP of the rectum and intestines was also lower, and the NTCP of the bladder was not significantly different. Therefore, it is speculated that 60Co may have a better protective effect on OARs without reducing the dose at the CTV. In addition, 60Co is cost effective, so the use of 60Co as an alternative to 192Ir for IC-ISBT it feasible and advantageous.

## Data Availability

The data used during this study are available from the corresponding author on reasonable request.

## Abbreviations

HDR: High dose rate
IC-ISBT: Intracavitary-interstitial brachytherapy
OARs: Organs at risk
CTV: Clinical target volume
EBRT: External beam radiotherapy
TPS: Treatment planning system
HIPO: Hybrid inverse planning optimization
DTGR: Dwell time gradient ratio
BED: Biologically effective dose
EQD2: Equivalent dose in 2 Gy/f
EUBED: Equivalent uniform biologically effective dose
EUD: Equivalent uniform dose
TCP: Tumor control probability
NTCP: Normal tissue complication probability
